# Recent Advances in the Treatment of Bone Metastases and Primary Bone Tumors: An Up-to-Date Review

**DOI:** 10.3390/cancers13164229

**Published:** 2021-08-23

**Authors:** Adrian Emilian Bădilă, Dragoș Mihai Rădulescu, Adelina-Gabriela Niculescu, Alexandru Mihai Grumezescu, Marius Rădulescu, Adrian Radu Rădulescu

**Affiliations:** 1 “Carol Davila” University of Medicine and Pharmacy, 050474 Bucharest, Romania; adrian.badila@umfcd.ro (A.E.B.); Dragos.radulescu@umfcd.ro (D.M.R.); radu_radulescu@umfcd.ro (A.R.R.); 2 Department of Orthopedics and Traumatology, Bucharest University Hospital, 050098 Bucharest, Romania; 3 Faculty of Applied Chemistry and Materials Science, University Politehnica of Bucharest, 060042 Bucharest, Romania; adelina.niculescu@stud.fils.upb.ro (A.-G.N.); agrumezescu@upb.ro (A.M.G.); 4 Research Institute of the University of Bucharest—ICUB, University of Bucharest, 050657 Bucharest, Romania; 5 Academy of Romanian Scientists, 3 Ilfov Street, 50044 Bucharest, Romania; 6 Department of Inorganic Chemistry, Physical Chemistry and Electrochemistry, University Politehnica of Bucharest, 1-7 Polizu St., 011061 Bucharest, Romania

**Keywords:** bone tumors, bone cancers, targeted therapies, drug delivery, bone substitutes, tissue-engineered scaffolds

## Abstract

**Simple Summary:**

Bone represents a common metastatic site for patients with advanced cancers while also being associated with rare but challenging primary tumors. As the treatment of bone sarcomas and metastases encountered a slowdown in its development, complementary and alternative strategies to conventional therapies started being investigated. In this respect, this paper proposes a comprehensive path, beginning with the types of bone tumors and current treatment options, and further detailing the recent advancements in the field, including adjuvant therapies, targeted drug delivery, novel bone substitutes, and multifunctional tissue-engineered scaffolds with synergic properties.

**Abstract:**

In the last decades, the treatment of primary and secondary bone tumors has faced a slow-down in its development, being mainly based on chemotherapy, radiotherapy, and surgical interventions. However, these conventional therapeutic strategies present a series of disadvantages (e.g., multidrug resistance, tumor recurrence, severe side effects, formation of large bone defects), which limit their application and efficacy. In recent years, these procedures were combined with several adjuvant therapies, with different degrees of success. To overcome the drawbacks of current therapies and improve treatment outcomes, other strategies started being investigated, like carrier-mediated drug delivery, bone substitutes for repairing bone defects, and multifunctional scaffolds with bone tissue regeneration and antitumor properties. Thus, this paper aims to present the types of bone tumors and their current treatment approaches, further focusing on the recent advances in new therapeutic alternatives.

## 1. Introduction

Bone is a highly active and dynamic connective tissue, which provides vital organ protection, mechanical support, locomotion, and structural body framework [1,2]. Bone has high functional stability and regeneration potential, but, in some cases, the self-healing capacity of the bone tissue is impeded in critical-sized defects caused by trauma, tumor, or infection [3,4]. In particular, bone represents a common metastatic site in cancer patients [5] and is also associated with the development of rare malignant primary tumors [6,7]. Both primary and secondary bone tumor treatment is challenging, as conventional therapies (e.g., chemotherapy, radiotherapy, surgical resection) face difficulties such as drug resistance and disease recurrence [6,8,9,10]. In this regard, considerable attention is directed to researching, developing, and implementing better bone cancer treatments [11].

In this respect, several complementary therapies started to be used in clinical practice [12]. Adjuvant therapies like cryoablation, laser ablation, argon beam coagulation, photothermal therapy, immunotherapy, and more have been employed with different success rates [13]. Despite the continuously increasing number of neoadjuvant modalities, the choice of optimum treatment remains challenging, as each of these therapies has both advantages and disadvantages [14]. Another promising approach is the development of improved drug administration options. Particularly, the delivery of desired compounds via micro- and nanostructured carriers has gained tremendous interest in recent years. It aids in the bioaccumulation of drugs at the target site, enhances therapeutic efficacy, and reduces systemic side effects [9,15].

When critical-sized defects result from bone weakening due to metastases or after surgical resection of tumors, the restoration of skeletal segment integrity requires clinical intervention [5,16,17,18]. In this respect, several approaches have gained interest for bone reconstruction (e.g., bone grafts, synthetic bone substitutes, tissue-engineered scaffolds). This problem is a hot topic of medicine, material science, and nanotechnology interdisciplinary research. 

Therefore, this paper proposes a comprehensive approach to bone tumors, detailing their types, current treatments, adjuvant therapies, drug carriers and formulations, and bone replacement options, aiming to set a clear context for future research in this field.

## 2. Bone Tumors

Bone tumors can be mainly divided into primary bone tumors (sarcomas) and secondary bone tumors (metastases). The most common sarcomas are osteosarcomas, chondrosarcomas, and Ewing sarcomas, accounting for more than two-thirds of such malignancies [10]. In what concerns secondary bone tumors, they can occur due to various types of advanced cancers [5]. Therefore, a detailed presentation is made in the following subsections to have a deep understanding of the differences between these types of bone tumors. 

### 2.1. Sarcomas

Bone sarcomas are tumors of mesenchymal origin [10,19]. They are primary bone tumors that emerge in a rich cell population environment due to the close interaction between tumor cells and local microenvironmental cell types, such as mesenchymal stem cells, cancer-associated fibroblasts, osteoblasts, osteocytes, osteoclasts, chondrocytes, or immune infiltrates [6,19].

**Figure 1:**
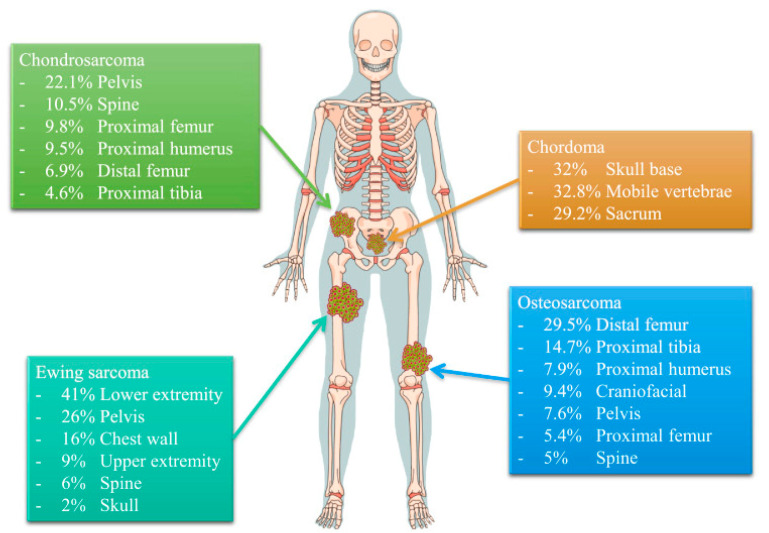
Anatomical distribution of common bone sarcoma. Reprinted from an open-access source [8].

The most frequent malignant primary bone tumors are, in this order, osteosarcomas (35%), chondrosarcomas (25%), and Ewing sarcomas (16%) [6,10,20] (Figure 1). 

These bone-originating tumors are a rare group of malignancies, comprising less than 0.2% of overall cancer diagnosis, with an adjusted incidence rate for all bone and joint malignancies around 0.9 per 100,000 persons annually [6,21]. Although they are uncommon, bone sarcomas are uniquely challenging, presenting high mortality rates and the overall burden of disease [8,10]. 

**Figure 2:**
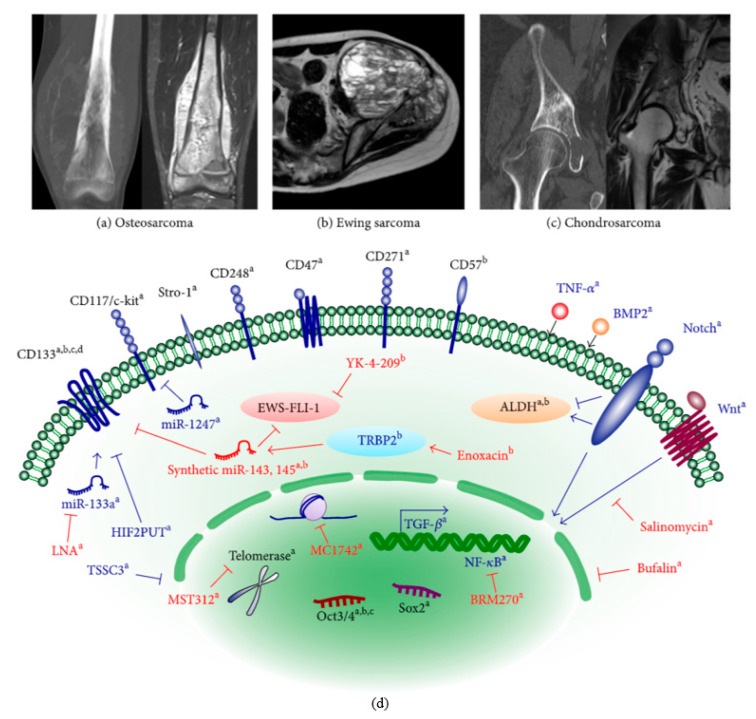
Bone sarcoma stem cells overview. (**a**) osteosarcoma; (**b**) Ewing sarcoma; (**c**) chondrosarcoma. (**d**) A broad spectrum of CSC markers (black) and the molecular mechanisms underlying CSC phenotypes (blue) have been documented for each sarcoma. Several anti-CSC compounds (red) have been preclinically tried to inhibit CSC phenotypes. Reprinted from an open-access source [20].

These tumors consist of heterogeneous cell populations, including cancer stem cells (CSC). CSC have the abilities of normal stem cells, especially their self-renewal and differentiation capacity. CSC can be more appropriately called “tumor-initiating cells” because it can produce all cells found in a particular tumor. [22]. Thus, CSC expresses a wide range of markers, varying according to tumor type and tissue of origin [20,22] (Figure 2).

#### 2.1.1. Osteosarcoma 

Osteosarcoma is the most prevalent primary malignant bone tumor, especially in adolescents and young adults [7,19,20]. Specifically, osteosarcoma was seen to occur predominantly in the second decade of life, during the adolescent growth spurt, with a peak incidence at 16 years for girls and 18 years for boys [23]. It is a highly aggressive tumor in which malignant cells produce pathological bone, having a natural tendency to metastasize [24]. This type of sarcoma is characterized by a disorganized bone structure, which can appear as a fine lacey trabecular pattern or as irregular clumps of osteoid, distinctly unlike normal bone formation. The most frequent osteosarcoma occurs in the juxta-epiphyseal regions of rapidly growing long bones [7]. 

Despite the adoption of aggressive, multimodal treatment schedules, the survival rate of osteosarcoma patients did not significantly improve over the last 30 years. The median 5-year survival rate is reported around 68%; however, it is highly dependent on the SEER stage, as follows: localized—74%, regional—66%, distant—27% [25,26,27]. Particularly, chemoresistance and preventing metastasis are still important challenges impeding successful osteosarcoma therapy [15,19].

#### 2.1.2. Chondrosarcoma

Chondrosarcomas are rare mesenchymal tumors, accounting for 10–20% of all malignant bone tumors. They have a cartilage-like appearance, being characterized by variously differentiated cells producing chondroid matrix [7,28]. Unlike osteosarcoma and Ewing sarcoma, this type of tumor is characteristic for adults, with over 70% of diagnosed cases present after the age of 40 [29].

Nonetheless, chondrosarcomas are a heterogeneous group of tumors, both from the morphological and clinical points of view. From this category of tumors, 80–90% are conventional chondrosarcoma, while about 10% are highly aggressive dedifferentiated chondrosarcoma. Conventional chondrosarcomas begin intramedullary growth, usually affecting the pelvis, femur, humerus, ribs, and ilium. However, femur, humerus, and pelvis are also the most frequent sites of dedifferentiated chondrosarcomas, which comprise chondroid and non-chondroid parts often looking like fibroblastic or osteoblastic tissue, indicating two types of mesenchymal differentiation in one tumor [28,29]. Moreover, chondrosarcoma has a high propensity to metastasize to distant organs, especially lungs [30]. 

The anatomic depth of these lesions often leads to a late diagnosis, which, coupled with the high resistance towards chemotherapy, radiation therapy, and even targeted approaches, results in poorer prognoses [7,14,28,31]. Hence, chondrosarcoma treatment options are quite limited, the most important option being represented by the surgical resection of such tumors [14,29,31].

#### 2.1.3. Ewing Sarcoma

Ewing sarcoma is the second most common malignant bone tumor in children and adolescents, with a peak incidence at 15 years [7,8,19,32]. It typically affects individuals in the first three decades of life, manifesting as a small, round, blue cell malignancy [7]. 

The conventional treatment approach of such tumors includes neoadjuvant chemotherapy, local treatment, and adjuvant chemotherapy. Local treatment may imply surgical intervention and/or radiation therapy. When possible, resection within healthy margins seems preferable to radiation therapy alone [32]. There is a five-year survival rate of 70–80% for localized Ewing sarcoma, but patient outcomes are significantly worse for those with pelvic involvement, large tumors, or incomplete tumor regression after chemotherapy [8].

### 2.2. Metastases

Bone represents a common metastatic site in patients with advanced cancer [5]. Bone metastases indicate a short prognosis, generating a major mortality rate. This happens because once tumor cells become home to the skeleton, the disease can rarely be cured. However, treatment is considered for alleviating pain and slowing its development [9,10,33]. Most bone metastases are caused by breast and prostate cancers, being more frequent than sarcomas, especially in the adult population [33,34]. 

**Figure 3:**
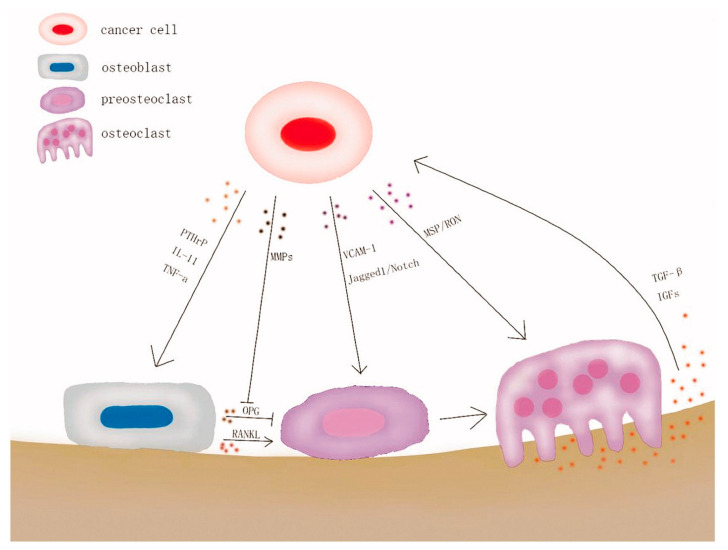
Graphical representation of the “vicious cycle” caused by cancer bone metastasis. Reprinted from an open-access source [36].

Skeletal sarcoma and metastases share the same tissue microenvironment and niches [10]. Evidence suggests that cancer cells can remain in a dormant state for decades in the metastatic niche before proliferation and metastasis formation. Nonetheless, tumor cells of bone metastases cannot destroy the bone directly, but they stimulate osteoclasts to degrade the bone ECM. Thus, a “vicious cycle” is promoted through the crosstalk between tumor cells and the bone microenvironment [5,34,35] (Figure 3).

Depending on the primary mechanism of interference with normal bone remodeling, bone metastases can be split into three categories: osteolytic, osteoblastic, and mixed (Table 1).

**Figure 4:**
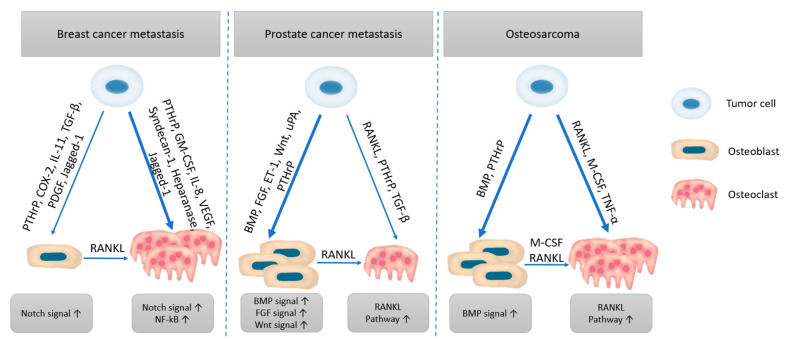
Comparison of bone tumor microenvironment in metastasis versus sarcoma. Created based on information from literature references [36,42,43,44,45].

As described in Table 1, bone tissue is an ideal site for many cancers to thrive [41]. The tumor cells that manage to metastasize to bone, hijack the physiological mechanisms controlling bone homeostasis. Consequently, an imbalance between osteoblasts and osteoclasts occurs [42]. According to the tumor type and tumor-derived factors, malignant cells act differently on the bone microenvironment [43]. For instance, prostate cells frequently secrete osteoblast-promoting factors (e.g., BMP, Wnt, ET-a, PDGF), whereas breast cancer cells commonly inhibit these pathways by producing soluble inhibitors while overexpressing osteoclast-inducing factors (e.g., PTHrP, IL-8, IL-11) [42]. Therefore, a clear understanding of the bone microenvironment and its interaction with tumor cells can help create targeted therapeutic strategies against different bone metastasis [44]. Moreover, distinguishing between the metastatic and sarcoma environments represents a promising starting point for approaching these diseases more specifically and efficiently [45] (Figure 4).

## 3. Current Treatments and Tumor Resistance

Bone tumors represent a medical challenge, especially due to their complexity, heterogeneity, aggressive behavior, and insignificant improvement in their treatment protocols for decades. The treatment of bone cancer has received considerable attention from scientists and clinicians, yet the therapeutic options for bone sarcomas have remained largely the same as in the late 1970s [6,10,11]. 

Treatment choice depends on various factors, such as the type of cancer and its characteristics, if the disease is localized or widespread, evidence of extraskeletal metastases, prior treatment history, symptoms, general state of health [33]. 

The most common approach for bone tumor eradication is surgical resection with adequate margins, combined or not with radiotherapy and/or chemotherapy [6,9]. Treatments can often shrink or slow the growth of bone metastases, providing symptomatic relief, but they are not curative [9,33]. Specifically, tumors can recur after the initial treatment response, behaving more aggressively and presenting an enhanced resistance to systemic therapies. Chemoradiotherapy resistance is acquired as CSC becomes dormant, having increased DNA repair, decreased apoptosis, and interacting with their supporting microenvironment [22]. 

### 3.1. Chemotherapy

Neoadjuvant chemotherapy regimens are employed to produce tumor necrosis, decrease primary tumor size, and reduce the number and size of pulmonary metastases [46,47]. The current chemotherapy possibilities include the usage of doxorubicin, high-dose methotrexate, cisplatin, and ifosfamide [7]. All cytotoxic drugs act against tumor cells by disrupting the cell cycle by one or more processes. They are administered at repeated, regular intervals, known as treatment cycles, and the treatment schedule depends on the ability of healthy tissues to recover. Most chemotherapy regimens involve two or more such agents in order to generate more effective outcomes [48]. Through the multidrug approach, the survival rates are around 70% in patients with no evidence of metastasis at the time of diagnosis. In contrast, patients presenting metastases only have a survival rate of 20% [7].

Chemotherapy is generally not effective for treating chondrosarcomas due to multidrug resistance [7,49]. Some of the mechanisms underlying chemoresistance in this type of tumors are the expression of multidrug-resistance gene P-glycoprotein, the high abundance of the cartilaginous matrix, the expression of anti-apoptotic proteins from the B-cell lymphoma 2 (Bcl-2) family, and the induction hypoxia-inducible factor 1α (HIF1α) by the high active kinase (AKT and SRC) [29,50]. 

Intensified treatments with combinations of cytotoxic drugs have also failed to improve the cure rates of conventional high-grade osteosarcoma significantly. The main cause is the inherent or acquired drug resistance, which, coupled with metastasis development, causes the dismal prognosis of relapsed patients, for whom cure probability is at most 20–25% [15,51]. 

### 3.2. Radiotherapy

Radiotherapy is a physical modality of destroying cancer cells. The applied radiation is called ionization radiation as it forms electrically charged particles and deposits energy in the cells it passes through. The high-energy radiation can directly kill tumor cells or damage their genetic material, impeding further division and proliferation [52].

Radiation therapy can be successfully employed as a palliative intervention to maintain and improve a patient’s quality of life, reduce analgesic requirements, and maintain or ameliorate skeletal function [53]. Besides, radiotherapy was seen to be effective as adjuvant therapy in unresectable or incompletely resected tumors, reducing local bone pain and inflammation [49]. In addition, radiotherapy may be used as the primary local control modality or combined with surgery for treating Ewing’s sarcoma [7].

In contrast, chondrosarcomas are considered radioresistant tumors, but the resistance mechanism has not been fully elucidated yet. Nonetheless, radiation therapy is used for metastatic disease as symptomatic treatment in selected cases after incomplete resection or unresectable tumors in difficult-to-reach anatomical sites [29,54]. 

In recent years, promising therapeutic options have risen from the combination of radiotherapy and ablative approaches, such as radiofrequency ablation, high-intensity focused ultrasound, and cryoablation [53].

### 3.3. Surgical Management

One of the most common strategies for bone cancer therapy is surgical intervention [55]. In malignant tumors, the surgical margins have to be wide or radical to reduce the risk of local recurrence, meaning that all the resected tumors must be covered by a layer of normal tissue [56]. 

The treatment protocol in osteosarcoma includes surgical resection of both primary tumor and bony metastasis, the surgical margin, reconstruction, and adjuvant therapy plan being further delineated by the subtype of osteosarcoma. Similarly, surgical management of Ewing sarcoma implies wide resection of lesions in the appendicular skeleton and selected resection for lesions in the axial skeleton [7]. The surgical treatment strategy for metastatic bone disease is also dependent on the residual estimated life expectancy, allowing the orthopedic surgeon to choose patient-specific surgical solutions [57].

Despite achieving high local tumor control rates, wide resection is associated with significant impairment of function due to the violation of the adjacent joint and formation of bone defects [13,55]. Hence, surgery is only indicated for fractures of long bones and hip joints, if the spinal cord is involved, or if peripheral nerve compression is observed [33]. Moreover, few bone tumor cells may remain around defects, potentially increasing and leading to cancer recurrence [55].

## 4. Recent Approaches against Bone Tumors

The two main challenges of bone tumor therapy are repairing large bone defects and eradicating all possible residual tumor cells [11]. Even with currently available aggressive treatments (e.g., extensive surgical resection, chemotherapy, radiotherapy), the outcomes have not considerably improved over the past few decades for osteosarcoma or Ewing sarcoma patients [8,58].

In this respect, there is an imperative need to find better treatment strategies and overcome the limitations of conventional therapies either by replacing them or working in tandem towards reaching higher survival rates and avoiding disease recurrence. 

### 4.1. Adjuvant Therapies

Adjuvant therapies can be employed for decreasing the high rates of local recurrence after curettage, consequently avoiding the need for extensive resection and reconstruction [13,59,60]. Various techniques and local adjuvants have been researched and used in clinical practice with different degrees of success. 

Out of these procedures, radiofrequency ablation is the most widespread and most thoroughly analyzed thermoablative technique [61]. Radiofrequency ablation is considered reliable, safe, minimally invasive, and effective for controlling bone tumors and relieving pain, having only a low rate of major complications related to secondary bone fractures [62,63,64,65]. It acts upon solid tumors by frictional heating produced when ions in the target tissue attempt to follow the changing directions of a high-frequency alternating current [66]. Subsequently, the Joule effect destroys adjacent tumor tissue. The associated dehydration results in increased tissue resistance during ablation, thus restricting energy application and limiting the expansion of the ablation area [61,67]. However, it should have proceeded with care as tumors larger than 3 cm and previous radiation therapy represent risk factors for complications [62].

Cryoablation is an adjuvant therapy that has been associated with a median local tumor recurrence rate of 11.2% in a large variety of benign–aggressive and malignant bone tumors [13]. It supposes the cooling of tumor tissue to −20 °C and transferring of energy from the inserted probe to the surrounding tissue through conduction and convection [61]. By means of ultrasound, magnetic resonance, or computed tomography guidance, the ablation zone can be accurately identified, thus helping the operator know exactly which anatomic structures are susceptible to thermal injury [68,69,70,71]. Cell death occurs in the immediately adjacent tissues to the probes through intracellular ice formation and subsequent cell destruction, while, at a further distance, there is a gradual cooling that causes osmotic differences across the cell membrane, secondary cellular dehydration, and death. Thus, cryoablation is considered effective against painful primary and secondary bone neoplasms [69]. However, it is a procedure with many complications, particularly due to peripheral bone necrosis and cold injury to the surrounding soft tissues. Moreover, the procedural complexity and high costs do not favor this adjuvant therapy [13]. 

Microwave ablation is another effective method for treating and achieving short- and mid-term pain relief for painful osteoid osteoma and malignant bone tumors, respectively [72,73,74]. During this procedure, a generator is utilized to produce a high-frequency electromagnetic field at the tip of the antenna inserted into the tumor [61,75]. Thus, an interaction occurs between the applied energy field and the intrinsic water dipoles in the adjoining tissue. Water molecules have the tendency to arrange along the energy field, their rotation generating kinetic energy, frictional heat, and eventually coagulative necrosis [61,76]. Thus, the procedure simplifies the surgery process, reduces the operation time, and decreases complication rates [61,73]. Besides, microwave ablation does not require grounding pads. The risk of thermal skin injury is minimized. Several antennae can be simultaneously inserted to achieve additive overlapping ablation areas, and there is no contraindication in patients with metallic implants. Nonetheless, the few disadvantages it presents should not be overlooked. Some of this technique’s drawbacks are the less distinct ablation zone margins than those achieved with radiofrequency ablation and cryoablation and the potential to overheat surrounding tissues that limit its use in the treatment of vertebral metastases [76].

Laser ablation is an alternative hyperthermal complementary procedure for the treatment of bone tumors. It uses optical fibers to transmit infrared light energy into a tumor to induce rapid heating of the targeted tissue, thus producing protein denaturation and consequent coagulative necrosis [61,76,77]. Image-guided laser ablation is considered an effective and safe debulking method that provides considerable pain relief in patients with bone metastases that do not respond to standard treatments [78]. The benefits of using this procedure are the predictable size of necrosis, lack of need to use neutral electrodes, lack of interaction with medical devices (e.g., stimulators, pacemakers, metallic implants), and low cost of disposable laser fibers. On the other hand, the main shortcoming is represented by the small size of the ablation zone for single fiber, limiting the utility of this technique in the treatment of large tumors [76,79]. 

High-intensity focused ultrasound (HIFU) is considered a revolutionary non-invasive technique that combines ultrasound energy with thermal feedback. Specifically, an ultrasound beam is focused on the lesion, and the generated heat induces coagulative necrosis and cell death [61,76,79]. MRI-guided HIFU has been FDA- and European Union-approved for pain palliation and local tumor control of osseous metastases (Table 2). Its main advantages are precise treatment planning, real-time monitoring of ablation zone with MR thermometry, continuous temperature mapping, and immediate therapy assessment. Nonetheless, the procedure is quite time-consuming due to the relatively small resulting ablation area, and the use of needles for the implementation of thermal protection may result in imaging artifacts and inhomogeneous attenuation of the ultrasound beam [61,76].

Another possibility is using phenol as a local adjuvant, a straightforward alternative with a low risk of procedural complications. It works on the principle of denaturation and coagulation of cellular proteins and DNA damage, thus inducing tumor cell necrosis. Phenol is considered an effective and safe adjuvant that can destroy about 1–1.5 mm of tumor tissue [13,80,81]. In addition, with increasing concentration, phenol can be bacteriostatic, bactericide, cytotoxic and nonselective, and local anesthetic [59]. Nonetheless, tissue penetration of phenol is poor, limiting necrosis to the superficial cell layers of the bone tumor [13]. Similarly, ethanol can also be used as a local adjuvant, offering a compromise between higher toxicity adjuvants and no chemical adjuvant at all [81,82]. 

Polymethyl methacrylate (PMMA) can also be employed in tumor management, as it was assumed that its heat of polymerization and direct toxic effect of monomers could be a mechanism of adjacent cellular toxicity [13,59,83]. However, it is not considered an adjuvant for local tumor control, but rather a mechanical reinforcement of the tumoral cavity. This is due to PMMA’s stiffness, which plays a role in the evolution of degenerative changes, especially when this material is overlaid or positioned close to the articular cartilage [13,81].

Argon Beam Coagulation (ABC) can also help in bone tumor treatment [7,83]. For decades, this technique has been known as a method for surface controls during major open surgical sites, using the same principles as a standard electrosurgical generator, but without the need for direct contact [84]. ABC supposes the delivery of unipolar electrical current through a fine beam of inert argon gas. Through the conduction of high-frequency electrical energy to the grounded tissue, immediate desiccation, coagulation, and shrinkage of the surrounding tissue are produced. Compared to other techniques, ABC is easier to manage, being considered a safe and reasonable adjuvant therapy with low recurrence rates [13,81].

Recently, photothermal therapy (PTT) has also attracted interest in cancer ablation. It is considered promising for tumor treatment especially due to its minimally invasive nature and specific spatiotemporal selectivity. Besides, PTT is economically viable, allows fast recovery, and prevents damage to the non-target regions [11,55,85]. For instance, iron nanoparticles present a photothermal effect while releasing Fe ions capable of H_2_O_2_ decomposition inside the tumor. Thus, reactive oxygen species are produced, which further damage lipids, proteins, and DNA, being harmful to tumor cells [11]. Such synergistic photothermal and photodynamic therapies can improve the tumor therapeutic effect compared with the monomodal therapies [11,86]

Another promising option in treating bone cancer is represented by immune-modulating therapies [29,87]. Macrophages first attack sarcoma cells; then, dendritic cells capture tumor-associated antigens at the tumor site and present them to T cells within the lymph node; next, T cells return and kill tumor cells. This antitumor immune response is regulated by the immune checkpoint mechanism. The working principle of immune therapy consists of attenuating the immune inhibitory molecules tumor cells employ to protect themselves from immune cells within the tumor microenvironment. In this respect, immune checkpoint inhibitors have attracted considerable attention for treating bone sarcoma and will continue to gain scientific interest as ongoing clinical trials begin to report on their results (Table 3). In particular, the combination of several immune checkpoint blockade treatment regimens has been proven better than single checkpoint blockade therapy [8,87].

### 4.2. Drug Delivery

As drug resistance is a common challenge in treating bone tumors, special attention has been attracted to developing better delivery systems to improve chemotherapy outcomes. To avoid using small systemic dose or large local dose of plain chemotherapeutics, nanocarrier systems with controlled drug release can be employed. Particularly, bone-targeted drug delivery systems aim to concentrate drugs in tumor sites, protect drugs from rapid clearance, prolong their circulation times, enhance therapeutic efficacy, and reduce the systemic adverse effects [9,15,88,89]. 

Bone-targeting carriers combined with the desired drugs or bioactive agents have been extensively investigated to enhance bone healing, while minimizing off-target effects [58,90]. In this respect, recent advances in developing both carrier systems and drug formulations are further presented.

#### 4.2.1. Carrier Systems

**Figure 5:**
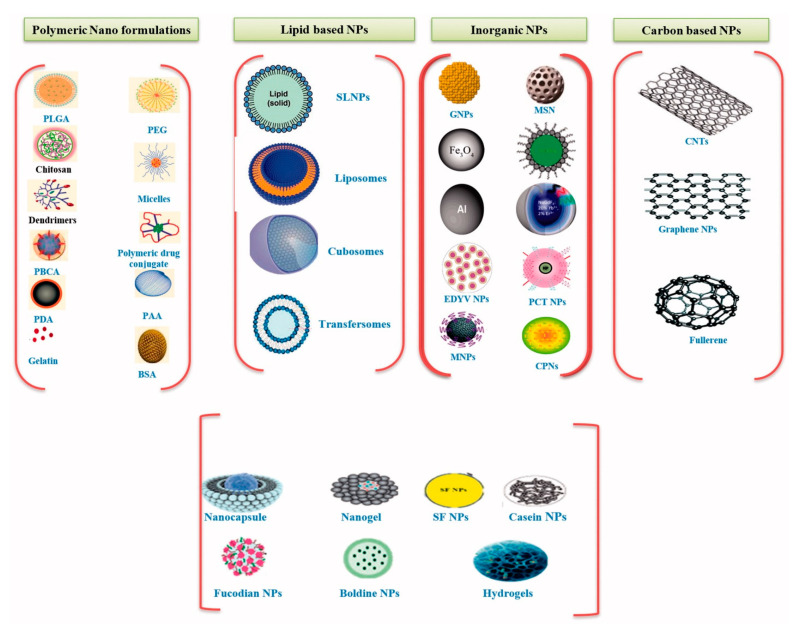
Various nanocarriers for anticancer drugs. Reprinted from an open-access source [94].

To overcome the limitations of systemic administration of drugs, micro-, and nano-sized vehicles have been developed to treat bone pathologies that exhibit specific affinity for bone [91]. Targeting delivery of drugs to the bone may enhance the treatment efficacy and reduce the quantity of administered drugs [92]. The carrier system should be biocompatible, non-immunogenic, and inert to the normal bone healing events [90]. In this regard, several organic and inorganic materials have been investigated as possible carriers for bone tissue regeneration in the past years [3,93] (Figure 5). 

Natural and synthetic polymers have been extensively researched for delivering drugs to target tissues [91,95]. Natural polymers, including chitosan, gelatin, collagen, fibrin, hyaluronic acid, silk, glycosaminoglycans, and alginate, are considered advantageous as carriers due to their inherent biocompatibility and bioactivity [3,90,96]. 

Chitosan is a deacetylated derivative of chitin that can enhance the absorption of hydrophobic macromolecular drugs when used as a carrier. Nanoparticles of this natural polysaccharide can extend the encapsulated drug’s activity by holding the therapeutic agent in closer proximity to the site of action due to its mucoadhesive cationic nature, thus facilitating trans-mucosal chemo-drug delivery [9,94,97,98,99,100]. Moreover, chitosan’s surface modification ability can be exploited towards obtaining new biological properties [101]. For instance, chitosan nanoparticles can be functionalized with sulfate groups to obtain a heparin-like polysaccharide structure, which can successfully link to the basic amino-acid stretches of BMP-2 [9]. Known for its controllable non-immunogenicity, controllable biodegradability and commercial availability, gelatin has also attracted attention to the delivery of biomolecules. Both micro- and nanosphere-based gelatin gels were shown effective in the sustained release of growth factors, the kinetics depending on the size of spheres with the same crosslinking density [102]. Moreover, the modification of gelatin and its combinations with other biomaterials have demonstrated the versatility of these systems, which can be exploited for developing ideal carrier systems that enable specific, targeted drug release [103]. Collagen structures can also be involved in the local delivery of osteoinductive differentiation factors [104]. Its flowability, injectability, biocompatibility, and network-like structural nature make collagen suitable for carrying the desired compounds to the target tissue [95,105]. Similarly, fibrin glue can act as a vehicle for drug and factors delivery, as these agents can be loaded in the gel by impregnation, covalent linkages, or affinity-based systems [106]. A similar release behavior is also noticed for alginate hydrogels, representing a promising growth factor delivery strategy for repairing challenging bone injuries [107]. 

Synthetic polymers also have properties of interest for drug delivery, such as defined chemistry, ease of modification, tunable porosity, and degradation time [90]. Particularly, polymer nanoparticles could be ideal candidates for cancer therapy due to their efficacy, simple elaboration, design, and wide structural variety [94]. Most commonly used synthetic polymers are polylactic acid (PLA), polyglycolic acid (PGA), polylactic-co-glycolide (PLGA), poly(ε-caprolactone) (PCL), poly-p-dioxanone, and copolymers consisting of glycolide/trimethylene carbonate [3]. PMMA can also find use as a carrier of antineoplastic and antiresorptive agents. Nonetheless, currently available studies of this material have only been performed on animal models, with no research being published on human subjects [13]. Polymeric micelles can also be a transportation alternative for overcoming drug resistance [15]. While the core can incorporate poorly water-soluble agents, the shell can stabilize the micelles in the aqueous environment and can be modified with stimuli-responsive or tumor-targeting moieties [93]. Other advantageous properties of polymeric micelles are their low molecular weight, maximum encapsulation of drug, lower critical micelle concentration, and improved and higher drug accumulation at the targeted tumor site [94]. Dendrimers also represent a promising class of polymeric drug delivery systems for chemotherapeutic agents and theranostics due to their hyper-branched nanostructure, biocompatibility, and multifunctionalization. Dendrimers allow the encapsulation of drugs either in their internal core by noncovalent bonds or by covalent conjugation with their surface functionalities [93,94,95,108]. 

Other organic delivery vehicles are lipid-based nanoparticles, which have great potential as chemotherapeutics carriers due to their biocompatibility and low toxicity [9]. Particularly, liposomes have attracted special attention as hydrophobic drugs can be entrapped in their lipid bilayer, while hydrophilic drugs can be encapsulated in their aqueous core. Liposomes can be used to enhance the intracellular accumulation of the transported drug into the target cells and reduce the negative side effects of standard chemotherapy [49,93,94]. Moreover, these nanocarriers allow surface modification towards obtaining multifunctional particles. For instance, glutamic hexapeptide-folic acid-modified liposomes have been shown excellent targeting activity to metastatic bone cancer, as they successfully delivered paclitaxel to bone tumors [92]. 

Inorganic nanomaterials, including metallic nanoparticles, mesoporous silica nanomaterials, carbon-based nanomaterials, and bioactive glasses, can also be employed in the delivery of chemotherapeutics required in bone tumor therapies [93]. Their magnetic and optical properties, chemical and biological inertness, physical stability, and ease of surface functionalization make inorganic nanoparticles promising for biomedical imaging and targeting cancer cells [94]. In this respect, pure metallic particles of gold, silver, and copper, or metallic compounds, such as oxides and Mxene, have been reported to be useful carriers of drugs and genes for treatment of bone sarcomas [93]. Mesoporous silica nanoparticles (MSNs) also have several characteristics that make them attractive as targeted delivery vectors. Specifically, MSNs can deliver antitumor drugs in a targeted manner and release them on demand to increase cellular uptake without any premature release before reaching the target site [9]. Carbon-based nanomaterials, including carbon nanotubes, graphene oxide, mesoporous carbon, and carbon dots, have drawn considerable attention and have been extensively studied for cancer therapy due to their good physicochemical properties. They have an easily modified surface, excellent photothermal conversion ability, supramolecular π-π stacking, and high adsorption ability [93,109]. Another category of inorganic delivery vehicles is represented by bioactive phosphate-based glasses and ceramics, like calcium phosphates, hydroxyapatite, and β-tricalcium phosphate, which recapitulate aspects of the native mineral environment [90,110]. These nanocarriers are considered promising for bone therapy applications as they are biocompatible, biodegradable, non-immunogenic, pH-sensitive, and easily modifiable, also showing to be preferentially accumulated in bone tissues [93].

#### 4.2.2. Carried Agents

Several drugs and factors have been studied for their potential in enhancing bone tissue healing [9]. One of the most investigated agents is bisphosphonates, which are analogs of pyrophosphate, a natural inhibitor of bone demineralization [33,111]. These small-molecule drugs have a short half-life in circulation, requiring high doses and long-term repeat use, but they can achieve strong penetration ability and uniform distribution in bone cells [112]. Bisphosphonates ameliorate pain, reduce fracture, and display antimyeloma and antitumor effects activity with prolonged overall survival registered for various malignancies [33]. 

Denosumab is another agent of interest in managing bone tumors. It is a human monoclonal antibody that inhibits RANKL, thus preventing the development of osteoclasts. Denosumab has the ability to prevent or delay fractures’ occurrence in patients with bone metastases, being safe to administer even to individuals with impaired renal function [33,95,113,114,115]. It has been shown to significantly reduce the risk of skeletal-related events, with superior performance to zoledronic acid (a drug from the class of bisphosphonates) [95,116]. However, unlike bisphosphonates, denosumab cannot accumulate in the bone, reversing its effect after treatment discontinuation [33,36]. 

Cisplatin is a first-line chemotherapeutic in bone sarcomas [117]. Despite being a potent antineoplastic and cytotoxic drug for cancer cells, many patients experienced severe side effects and relapse due to drug resistance [118,119]. To avoid these drawbacks, cisplatin can be loaded in various nanoparticles that ensure its safe delivery to targeted tissues, enhance its activity towards tumor cells, and significantly reduce its systemic toxicity [117,120].

Doxorubicin is also an important drug in cancer management [121]. Nonetheless, it can face resistance from tumor cells, thus hampering the efficacy of the treatment [109]. Moreover, this drug causes severe side effects, such as myelosuppression, heart failure, and hepatic toxicity, especially when used in high doses needed for tumor growth control [122]. For these reasons, encapsulating doxorubicin in nanocarrier systems is an attractive approach, which safely delivers the anticancer drug to the bone, while increasing its toxicity and intracellular retention in tumor cells [9,109,121,122,123].

The inclusion of paclitaxel in nanoparticles is another viable strategy for bone cancer treatment [124,125,126]. By itself, paclitaxel is a potent anticancer drug, which, however, can lead to serious adverse effects [9]. Besides, it presents poor water solubility and has no bone surface targeting specificity [124]. These issues can be solved by loading the drug into biocompatible nanosystems with bone-affinity functionalization [92,124,125]. Such delivery approaches have been shown to effectively suppress tumor growth in the bone, improve the bone accumulation of paclitaxel, and hold great promise in treating drug-resistant cancers [125,126].

Other chemotherapeutic agents that have attracted interest for nanoparticle encapsulation include, but are not limited to, rapamycin, gentamicin, chloroquinone, etoposide, arsenic trioxide, pirarubicin, curcumin, ifosfamide, gemcitabine, and methotrexate [7,127,128,129,130,131,132,133,134,135].

Oncolytic virus therapy is a novel approach that has emerged as an alternative to conventional chemotherapeutics [127,136,137]. These viruses are genetically engineered to selectively replicate in and kill exclusively tumor cells [138]. Several preclinical studies have shown encouraging results in the systemic administration of oncolytic viruses against osteosarcoma [139,140,141]. Nonetheless, few obstacles were reported to hinder the outcomes of oncolytic virus therapy. These limitations comprise the immune system’s role, the effect of viral tropism toward the liver, and the physical barriers, including tumor extracellular matrix and limited extravasation of oncolytic viruses [127,142]. Thus, a better strategy is to use nanocarriers to deliver oncolytic viruses to the tumor site [127,143]. By using liposomes, polymers, biodegradable copolymers, and nanoparticles, the effects of virotherapy can be much enhanced due to increased concentrations at tumor sites, shielding within the circulation, and facilitated tumor targeting and cell entry [142].

As the use of a single drug-based treatment may be unsatisfactory, the use of combinatorial therapies has emerged as a promising strategy in treating bone tumors [144]. In this respect, research started to focus on developing synergic formulations of anticancer drugs and their co-delivery using various types of nanocarriers [130,131,132,135].

## 5. Bone Reconstruction Following Tumor Resection

Unlike other tissues, bone has a great capacity to regenerate. However, massive bone defects may be generated after curettage or aspiration of bone tumors, requiring clinical intervention. The restoration of the continuity and integrity of skeletal segments represents a challenge for orthopedists and a hot topic of research [1,2,5,16,17,18]. In this respect, several approaches have gained interest for bone reconstruction following tumor resection.

### 5.1. Bone Grafts

Even though bones have a high potential for self-regeneration, the efficacious repair of large bone defects still requires the implantation of bone grafts [9]. Several types of grafts can be employed in bone reconstruction: autografts, allografts, xenografts, and synthetic bone grafts (described in more detail in the next section) [16,145].

The gold standard for reconstructing bone defects is still represented by autologous grafts (also known as ‘autografts’ and ‘autogenous graft’) [146,147,148,149,150]. Autografts are harvested from the patient, being ideal in many situations [145]. Properties like osteoinduction, osteoconduction, and total histocompatibility of autografts make them suitable for bone regeneration, reducing the risk of immunogenic reaction and disease transmission [9,150]. However, intervening at healthy sites to collect autografts can produce donor-site morbidity, thus restricting this therapeutic approach [146,149,151]. Other drawbacks are represented by the limited amount of tissue that can be harvested, the need for additional surgery, and prolonged operative times [1,9,16]. 

To overcome the issue of quantity, bone allografts or xenografts can be used instead [150,152]. Allograft bone can be collected from either living or nonliving human donors and must be processed within a bone tissue bank [153]. Allografts are osteoconductive, little osteoinductive, not osteogenic, and present certain disease transmission risks [150,154]. However, in modern times, the risk of infection is diminished through graft treatment and sterilization. Nonetheless, these aspects increase the procedure’s costs and time, also affecting the bone graft’s mechanical properties [155]. A similar discussion raises for xenografts, except they are harvested from other species, such as bovine or porcine bone tissues [150,155]. In addition, implants of animal tissues may come in contradiction with some patients’ religious beliefs and ethical concerns about animal rights, leading to the refusal of such a procedure [150,156,157,158].

In an effort to avoid the issues associated with natural grafts, artificial grafts (synthetic substitutes) have been fabricated to repair critical-sized bone defects [1,16,147]. 

### 5.2. Bone Substitutes

In the last decade, synthetic bone substitutes have become a promising alternative to autografts, allografts, and xenografts [159]. As these bone grafts are synthetically manufactured, patients would be more likely to accept their use for surgical procedures [150].

An ideal artificial graft must be biocompatible and biodegradable while supporting osteoconduction, osteoinduction, and osteointegration [3,160]. Additionally, the grafting material should lack carcinogenicity, teratogenicity, be well-tolerated by the organism, bioresorbable, hydrophilic, non-antigenic, non-toxic, affordable, easy to manipulate, sterile or sterilizable, and have excellent biomechanical characteristics [18,161]. 

A chemical composition similar to natural bone is considered advantageous for artificial bone substitutes [146]. It has also been shown that physical characteristics, such as granule size, granular shape, and pore size, impact the tissue reaction response to a bone substitute, influencing their regenerative potential and expression of cytokines produced by monocytes [159]. Taking everything into account, various materials, including hydroxyapatite, calcium sulfate, calcium phosphate ceramics, and bioactive glasses, have been considered promising options for bone substitutes [150,155]. Several bone substitutes are already available on the market; three of them are briefly described in Table 4. Their crystallite size and specific surface area are consistent with the fact that Neobone^®^ (HAp) and Cerasorb^®^ (β-TCP) are sintered ceramics, while Cytrans^®^ (CO_3_Ap) granules are fabricated through a dissolution–precipitation reaction in an aqueous solution using a calcite block [146].

Another limb-salvage option is the endoprosthetic reconstruction using modular prostheses with either cemented or cementless fixations. This approach is considered reliable in periarticular tumor resections, offering component modularity, improved fixation, and good-to-excellent functional results. Moreover, cementless systems minimize the risk of failure, having favorable outcomes regarding infection and aseptic loosening [162].

Efforts have also been directed to combining bone substitutes with antibiotics to prevent potential infections at their implantation site. However, only a few such synthetic grafts are commercially available due to their challenging fabrication. Specifically, the major problem in combining antibiotics with bone substitutes is the exothermic polymerization reaction during material processing, which has fatal consequences on heat-sensitive antibiotics [3]. Alternatively, antibiotics can be loaded into ceramic-based substitutes to ensure their intraosseous delivery. Nonetheless, two drawbacks have to be considered: antibiotic resistance of certain bacterial strains and achieving antimicrobial activity without hindering osseointegration [163]. To avoid such issues, doping ceramics with metallic compounds with inherent antibacterial properties can replace the use of antibiotics. In this regard, metal ions, including copper, silver, iron, titanium, strontium, manganese, and zinc, have been doped successfully into bioceramic and hybrid scaffolds, enhancing their anti-infective properties [164,165,166,167,168,169,170,171,172,173,174].

### 5.3. Tissue Engineering Approaches

The field of tissue engineering allows the creation of smart multifunctional bone substitutes that use a porous structure (called scaffold) serving as a template for cell attachment, differentiation, proliferation, and tissue regeneration [1,9,149]. 

The choice of a suitable material for the scaffold is an essential step in designing tissue-engineered bone substitutes. The material should mimic the native extracellular matrix guiding resident stem cells to regenerate the functional tissue [16]. Thus, tissue-engineered scaffolds preserve the requirements of ideal synthetic bone substitutes plus the features needed for osteogenic differentiation and maturation of the included cells [9,149,175]. These bioscaffolds should be able to interact with the cellular component of the bone, have an adequate morphology for permitting vascular ingrowth and cellular transportation, and be biodegrade at a predictable rate [9,176,177]. 

Natural polymers can be employed to fabricate bone scaffolds due to their resemblance with native ECM, biocompatibility, and high osteoinductive properties. Such structures can be derived by cells or directly obtained from decellularized bone tissue. Nonetheless, synthetic polymers provide better control of porosity and physicochemical properties, being promising biomaterials for bone tissue engineering [9,16,178,179]. Intensive research has also been directed to scaffolds made of bioceramics and their composites, as they have the necessary properties for biological activity in regard to cell adhesion, migration, and proliferation. Historically, their inherently low fracture toughness and strength limited their use in load-bearing applications, but the currently known variety of bioceramics composition has allowed the adjustment of these materials’ mechanical features, bioactivity, and degradation rate [160,161,180,181]. Another possibility of producing scaffolds with tailored properties is developing composites containing different bioceramics and polymers in different ratios [161,178,179]. 

Moreover, several advancements were reported in the development of novel bone replacements from classic metallic materials. Metals, such as titanium alloys, cobalt-chromium-molybdenum alloys, and stainless steel, are conventionally employed in orthopedic surgery [182]. However, such materials have quite different elastic mechanical properties than bone tissue. For instance, commercially available pure titanium and titanium alloy (Ti6Al4V-ELI) present a modulus of elasticity between 100 and 115 GPa, while the elastic modulus ranges from 3 to 30 GPa for cortical bone and from 0.02 to 2 GPa for cancellous bone. In this respect, advanced additive manufacturing technologies have recently enabled the fabrication of fully porous metallic biomaterials that could mimic the native bone tissue mechanical behavior, while also offering a large surface-to-volume ratio that helps in bone regeneration [183,184,185]. 

**Figure 6:**
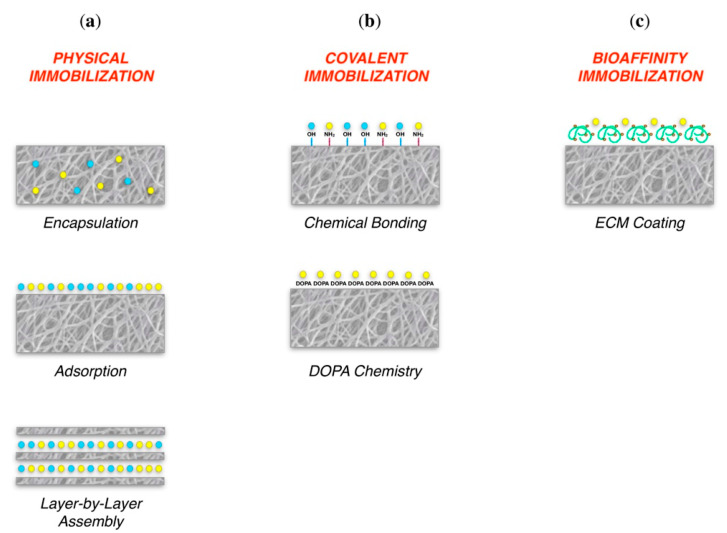
Graphical representation of functionalized scaffolds. (**a**) Physically immobilized bioactive molecules; (**b**) Covalently bound bioactive molecules; (**c**) Scaffold coated with ECM molecules. Reprinted from an open-access source [16].

To improve the inherent properties of the base material, bone scaffold surfaces can also be functionalized through several methods, such as coatings and immobilized molecules [3,16,186] (Figure 6).

The functionalization of the bone replacement with factors is a promising approach for attracting and stimulating cells from the surrounding host tissue after implantation, promoting osteogenic cells’ ingrowth, and forming a vascular network within the implant [149]. Growth factors for promoting osteogenesis and angiogenesis can be incorporated in scaffolds, while these structures can also act as stem cell carriers for accelerating bone repair [161]. One of the most potent osteoinductive growth factors (GFs) that can enhance the properties of the bone scaffold is bone morphogenetic proteins (BMPs) [3,176,187]. Other factors that can be added to bioscaffolds are PDGF, fibroblast GFs, vascular endothelial GFs, insulin-like GFs, TGF-β, IL-1, IL-6, and macrophage colony-stimulating factor [161,188].

Besides bone tissue regeneration, novel functional bioceramic scaffolds have also been endowed with tumor therapy ability. Hence, such innovative smart bone substitutes can repair the bone defects resulted after surgery, while also destroying possibly residual tumor cells. Specifically, the high temperature-induced by functional scaffolds produces irreversible protein denaturation, cell membrane damage, and late progressive apoptosis. Hence, they can serve as photothermal or magnetothermal agents locally and further promote new bone formation in vivo [55,189].

Moreover, biomaterials can act as local drug delivery systems. Synthetic scaffolds can incorporate antitumor, antibiotic, and anti-inflammatory compounds, ensuring their release in the target area, aiming to achieve a high local concentration, while maintaining the side effects at a minimum level. Therefore, treatment efficiency is enhanced, and bone function and integrity are restored [180,190,191].

## 6. Conclusions and Future Perspectives

To summarize, the complexity, aggressive progression, and lack of significant improvement in treatment protocols over the last decades still render bone tumors a medical challenge. Whether bone sarcomas or metastases of advanced cancers originated elsewhere in the organism, bone tumors require extensive attention and prompt treatment. For these reasons, special focus is directed towards improving conventional approaches by implementing complementary therapies, delivering drugs through carrier systems instead of systemic administration, and designing multifunctional scaffolds for repairing bone defects resulting from bone malignancies or tumor resection. Nonetheless, some of the presented therapeutic strategies have not reached the stage of clinical trials, requiring further investigations before moving to humans. Hence, more detailed studies are to be expected in the near future before the clinical implementation of innovative chemotherapeutic-loaded nanosystems. Moreover, novel pharmacological formulations can be more efficiently tested by assessing improved 3D cellular models (e.g., cancer-on-a-chip or organ-on-a-chip microfluidic devices), maintained in controllable microenvironments, and functioning as a whole organ. Other promising directions may emerge from research on bone turnover markers, predictive biomarkers, multimodality imaging, and co-delivery of multiple theragnostic agents.

To conclude, the recent advancements in the therapeutic strategies against bone tumors are encouraging, paving the way for the standardization of new treatments. Through the interdisciplinary approach of medicine, material science, and nanotechnology, bone metastases and sarcomas can be successfully fought against.

## Figures and Tables

**Table 1:** Bone metastases—types and characteristics.

Type of Bone Metastasis	Radiographic Appearance	Manifestation	Types of Cancer in Which It Appears	Producing Factors	Refs.
Osteolytic	Radiolucent areas located in the skull and proximal ends of long bones	Destruction of normal bone resulting in complications of bone pain, fracture, hypercalcemia, and nerve compression syndromes	Breast cancer Multiple myeloma Renal cell carcinoma Melanoma Non-small cell lung cancer Non-Hodgkin lymphoma Thyroid cancer Langerhans-cell histiocytosis	Parathyroid hormone-related protein (PTHrP) Interleukin (IL)-11 IL-8 IL-6 Receptor activator of nuclear factor-κ B ligand (RANKL) Bone-derived transforming growth factor-β (TGF-β) Connective tissue growth factor (CTGF)	[33,35,37,38,39]
Osteoblastic (or osteosclerotic)	Dense areas located to the axial skeleton and, particularly, in vertebral bodies and pelvis	Deposition of new bone with dysregulated bone resorption and bone formation	Prostate cancer Carcinoid Small cell lung cancer Hodgkin lymphoma Medulloblastoma	Platelet-derived growth factor (PDGF) Insulin-like growth factors Adrenomedullin Vasoactive peptide ET-1 PTHrP fragments	[33,35,38,39]
Mixed	Fuzzy aspect; a sclerotic rim of reactive bone, starting at the periphery and eventually involving the center of osteolytic lesions with continued healing is observed	Association of both osteolytic and osteoblastic lesions, or osteolytic and osteoblastic components in an individual metastasis	Breast cancer Gastrointestinal cancers Squamous cancers	Factors of both osteolytic and osteoblastic tumors	[33,35,40]

**Table 2:** Examples of HIFU clinical trials.

Clinicaltrails.Gov Identifier	Official Title	Purpose of the Study	(Estimated) Primary Completion Date
NCT01117246	“Pilot Study for the Treatment of Bone Metastases by High Intensity Focused Ultrasound Guided by MRI to Perform Pain Palliation”	Confirm the safety and effectiveness of MR-guided HIFU for pain palliation of skeletal metastases	July 2011
NCT01765907	“Antalgic Treatment of Painful Bone Metastases by US-guided High Intensity Focused Ultrasound (HIFU)”	Assess safety, including adverse and serious adverse events, local and systemic tolerance of HIFU in patients with bone metastasis	March 2014
NCT01964677	“Magnetic Resonance-Guided High Intensity Focused Ultrasound for Palliation of Painful Skeletal Metastases—a Multicenter Study”	Evaluate the effectiveness of Philips Sonalleve MR-HIFU device for the palliation of pain in patients with bone metastases	14 November 2016
NCT02618369	“Magnetic Resonance-Guided High Intensity Focused Ultrasound for Pain Management of Osteoid Osteoma and Benign Bone Tumors in Children and Young Adults”	Determine if MR-guided HIFU is safe and effective for alleviating pain associated with osteoid osteoma and other benign bone tumors in pediatric patients and adults (up 40 years old)	April 2018
NCT02349971	“Safety and Feasibility of MR-Guided High Intensity Focused Ultrasound (MR-HIFU) Ablation of Osteoid Osteoma in Children”	Examine the feasibility and efficacy of using MR-HIFU to ablate osteoid osteoma in children and young adults	3 October 2020
NCT03106675	“MR Imaging- Guided High Intensity Focused Ultrasound (HIFU) Therapy of Bone Metastases”	Evaluate the effectiveness of MR-guided HIFU in treating metastatic bone tumors	31 December 2021
NCT02076906	“Safety and Feasibility Study of Using MR-guided High Intensity Focused Ultrasound (HIFU) for the Ablation of Relapsed or Refractory Pediatric Solid Tumors”	Determine if MR-guided HIFU is safe and feasible for children, adolescents, and young adults with refractory or relapsed solid tumors	30 January 2022
NCT04658771	“Pivotal / Phase II Clinical Trial of Magnetic Resonance-Guided Focused Ultrasound (MR-HIFU) Treatment of Painful Osteoid Osteoma in Children and Young Adults”	Determine treatment safety and efficacy of MR-HIFU ablation of painful osteoid osteoma in children and young adults	30 January 2023
NCT04307914	“Focused Ultrasound and RadioTHERapy for Noninvasive Palliative Pain Treatment in Patients with Bone Metastases”	Evaluate the effectiveness and cost effectiveness of MR-HIFU (alone or in combination with EBRT) compared to EBRT alone	1 January 2024

**Table 3:** Examples of clinical trials involving immune modulation.

Clinicaltrails.Gov Identifier	Official Title	Purpose of the Study	(Estimated) Primary Completion Date
NCT02423928	“A Phase I Clinical Trial of Combined Cryotherapy and Intra-tumoral Immunotherapy with Autologous Immature Dendritic Cells in Men with Castration Resistant Prostatic Cancer and Metastases to Lymph Nodes and/or Bone Pre or Post Chemotherapy”	Evaluate the combined anti-cancer therapy response in patients with invasive castration resistant prostate cancer and radiologically verified metastases	16 August 2019
NCT03996473	“An Open-label, Multicenter, Phase 1/2 Study of Radium-223 Dichloride in Combination with Pembrolizumab in Participants with Stage IV Non-small Cell Lung Cancer”	Determine the safety and efficacy of the combination of radium-223 dichloride and pembrolizumab in patients with stage IV non-small cell lung cancer (NSCLC) with bone metastases who either have not received any systemic therapy for their advanced disease or have progressed on prior immunologic checkpoint blockade with antibodies against the programmed cell death protein-(ligand) 1 (PD-1/PD-L1)	14 April 2021
NCT03406858	“Phase II Trial of Immune Checkpoint Inhibitor with Anti-CD3 x Anti-HER2 Bispecific Antibody Armed Activated T Cells in Metastatic Castrate Resistant Prostate Cancer”	Study how well pembrolizumab and HER2Bi-armed activated T cells work in treating castration resistant prostate cancer that has spread to other places in the body, including bone	1 December 2021
NCT04516122	“Bone Loss in Cancer Survivors Receiving Adjuvant Immune Checkpoint Inhibitor Therapy”	Investigate the bone-related side effects caused by immunotherapy drugs	31 July 2022

**Table 4:** Comparison of physical properties of commercially available bone substitutes. Adapted from an open-access source [146].

	Bone Substitute	Neobone^®^ (HAp)	Cytrans^®^ (CO_3_Ap)	Cerasorb^®^ (β-TCP)
Property				
Crystallite size (nm)		75.4 ± 0.9	30.8 ± 0.8	78.5 ± 7.5
Specific surface area (m^3^/g)		1.0	18.2	0.06
CO_3_ content (%)		-	11.9	-
Bulk density (g/cm^3^)		0.47 ± 0.02	0.99 ± 0.03	0.72 ± 0.03
Porosity (%)		85.1 ± 0.5	68.7 ± 0.9	76.4 ± 0.8

HAp—hydroxyapatite; CO_3_Ap—carbonate apatite; β-TCP—β-tricalcium phosphate.

## Data Availability

Not applicable.
